# Hospitalisations Due to Dental Infection: A Retrospective Clinical Audit from an Australian Public Hospital

**DOI:** 10.3390/dj12060173

**Published:** 2024-06-06

**Authors:** Mafaz Ullah, Muhammad Irshad, Albert Yaacoub, Eric Carter, Stephen Cox

**Affiliations:** 1 Discipline of Oral Surgery, Faculty of Medicine and Health, University of Sydney, Sydney, NSW 2750, Australia; 2 Nepean Centre for Oral Health, Nepean Blue Mountains Local Health District, Kingswood, NSW 2747, Australia; 3 Khyber College of Dentistry, Peshawar 25000, Khyber Pakhtunkhwa, Pakistan; 4 Specialised Dental Center, Ministry of Health, Sakaka Aljouf 72345, Saudi Arabia; 5 School of Nursing and Midwifery, Western Sydney University, Penrith, NSW 2751, Australia

**Keywords:** dental caries, dental infection, odontogenic infection, hospitalisation, morbidity, Australia

## Abstract

The aim of this clinical audit is to evaluate the characteristics of dental infections requiring hospitalisations, which may help improve preventative and management policies. This study retrospectively evaluated the records of patients admitted to the Nepean hospital, Kingswood, New South Wales, Australia, due to dental infections between 2018 and 2019. A total of 102 patients, mostly in their thirties with equal gender distribution, were admitted with dental infections, presenting with pain (100%), swelling (99%), trismus (40.2%), dysphagia (27.4%), fever (21%) [>37 °C], tachycardia (24.8%) and tachypnoea (9.3%). Most patients (68%) presented on weekends, outside regular working hours, and public holidays. A total of 52.5% of patients had taken prior antibiotics. Dental caries, smoking, mental health issues, and illicit drug use were featured strongly. The majority of patients (56.4%) underwent treatment under local anaesthesia. The total length of hospital stay was 271 days (mean 2.7, SD 1.6). Augmentin was the most prescribed antibiotic. Complications were reported in 8.8% of the patients, primarily due to airway compromise. Dental infections leading to hospitalisations continue to be a burden on the healthcare system. A notable finding was that the presentations were primarily on weekends, outside regular working hours, and public holidays, and the majority required dental interventions under local anaesthesia. The provision of on-call emergency dental services may reduce potentially preventable hospitalisations and the length of hospital stay.

## 1. Introduction

Dental infection, often referred to as odontogenic infection, is an infection of the alveolus, jaws, or face originating from the bacterial invasion of endodontic or periodontal tissues [1]. The etiological factors include dental caries, periodontal infections, pericoronitis, failed endodontic treatments, dental trauma, severe dental attrition, and infected dental cysts. [2]. The preventable and treatable nature of these underlying causes emphasises the importance of timely dental interventions [3]. Without appropriate dental treatment, these conditions may lead to periapical and periodontal abscesses [4]. Advancements in dental science have facilitated the successful treatment of localised dental infections within outpatient settings in dental clinics [5]. However, if left untreated, dental infections can result in severe and potentially life-threatening infections, necessitating hospitalisation [6]. In a hospital setting, the management of dental infections often involves surgical intervention under general anaesthesia. This involves removing the infection source, performing surgical incisions with intraoral and/or extraoral drainage, and the administration of intravenous antibiotics and supportive care [7,8].

Dental infections resulting in hospitalisation pose a substantial risk to oral health-related morbidity and mortality and are a significant burden on public health in the form of potentially preventable hospitalisations [9]. Complications arising from dental infections encompass a wide spectrum, ranging from maxillary sinusitis and osteomyelitis to more severe conditions like Ludwig’s angina leading to airway compromise, including systemic complications such as septicaemia and septic shock [10]. Dental infections have been reported to be the most common cause of oral health-related preventable hospitalisations [11]. Studies have highlighted the considerable financial impact, with reported average hospital costs ranging from AUD 12,228 (Australia) to USD 47,835 (United States) per patient [12,13]. A study from the United States reported 61,439 hospitalisations attributed to dental infections with 2.96 days mean length of hospital stay and 66 deaths [14]. The cost of dental infection to humans in the form of serious morbidities and mortalities and potentially preventable hospitalisation is evident across the globe [8,9,11,15,16,17].

Recognising that dental caries and periodontal diseases are preventable and treatable conditions emphasises the importance of early dental interventions. Proactive oral health-related management strategies can mitigate complications and reduce the incidence of preventable hospitalisations [18]. A recent systematic review underscored the need for comprehensive data collection to guide evidence-based policies for the prevention and management of dental infections [11]. In line with this imperative, the current clinical audit is a preliminary study to evaluate the characteristics of patients presenting with dental infections requiring hospitalisations to the emergency department of a major public hospital in Australia to identify patterns that may help improve preventative and management policies.

## 2. Materials and Methods

### 2.1. Ethical Application

Human research ethics application (2020/ETH01100) was accepted on 14 June 2020. The site-specific assessment application (2020/STE01811) was accepted on 2 September 2020.

### 2.2. Patient Selection

This study retrospectively evaluated the records of patients admitted to the Nepean hospital, Kingswood, New South Wales 2747, Australia, for the management of dental infections for 12 months (July 2018–June 2019) in the pre-COVID period. Nepean hospital is a major public teaching hospital providing health services for the Nepean Blue Mountains Local Health District (NBMLHD). The study population included all the patients who presented to the emergency department of Nepean hospital with dental infections requiring hospitalisations during the study period. The study population predominantly represents patients from the NBMLHD. Key inclusion criteria included inpatients, without age restriction, presenting with dental infections or post-operative dental infections to the emergency department of Nepean hospital requiring hospitalisations during the study period. Key exclusion criteria included outpatients presenting with dental infections not requiring hospitalisations, inpatients and outpatients presenting with oral health-related emergencies other than dental infections, and non-oral health-related emergency patients presenting to the emergency department of Nepean hospital.

Patients were identified retrospectively from the Excel data provided by the emergency department of the Nepean hospital. Using the Excel option ‘sort and filter’, the data were screened for oral health-related presentations using various keywords. The keywords used for screening patients with dental infections requiring hospitalisations included teeth, tooth, dental, mouth, jaw, facial, neck, maxilla, and mandible combined with pain, odontalgia, infection, abscess and swelling, and hospital admission. Patient’s specific data were accessed on the electronic medical record software ‘Cerner Millenium Powerchart 2011’ using the patient’s specific medical record number (MRN). According to the International Classification of Disease, revision 11 (ICD-11), code DA09.6, dental infections leading to dental abscesses were identified from patient records which required hospitalisations.

### 2.3. Date Collection

Deidentified data related to outcome measurement was transferred to Microsoft Excel software (© 2024 Microsoft Corporation, Redmond, WA, USA) for further analysis. Data were collected for each individual patient for the following variables: date and time of presentation, age, gender, aboriginal and torres strait islander status, smoking, comorbidities, body mass index, clinical features, vital signs, facial space involvement, aetiology, jaw and teeth involvement, investigation, treatment, previous treatment, hospital stay, culture and sensitivity/microbiology, and outcome.

Demographic information was recorded upon presentation. Body mass index and clinical data at presentation included respiratory rate, oxygen saturation, pulse rate, blood pressure, mean arterial pressure, temperature, and random blood sugar. Medical, dental, and social histories and clinical examinations, including previous treatments, aetiology, pain, facial swelling, trismus, dysphagia, and facial space involvement, were recorded from the clinical notes. Orthopantomogram (OPG) was taken to investigate the aetiology and odontogenic origin of dental infection. Identification of cellulitis, frank abscess collections, and facial space involvement was confirmed by the radiologist’s report on contrast-enhanced computed tomography (CT) scans in selected cases. Blood tests, including white blood cell count, C-reactive protein, OPG, and contrast-enhanced CT, were recorded from the investigations.

Surgical treatments, type of antibiotics, length of hospital and intensive care unit stay, and complications were recorded from clinical and discharge notes. Procedures under general anaesthesia were performed at Nepean hospital theatre by consultants and registrars of the oral and maxillofacial, Plastics, and Ear Nose and Throat (ENT) departments. The Plastics and ENT clinicians performed incision and drainage only and referred patients to private dentists or the Nepean Centre for Oral Health (public dental clinic) for dental management. Patients treated under local anaesthesia had antibiotics commenced at Nepean hospital and were then transferred to Nepean Centre for Oral Health for surgical procedures. Patients were transferred back to the respective wards following procedures by general dentists or oral surgery registrars and discharged from the hospital. Some measurements were not recorded, and the total number reported in this study is presented as “*n*” in the data table.

### 2.4. Data Analysis

The data were rearranged in Microsoft Excel (© 2024 Microsoft) for each measurement, and subsequent analysis was performed utilising various functions. Following the analysis, the data were transferred into a table format in Microsoft Word. Additionally, graphs and figures were generated using Microsoft Excel. 

## 3. Results

### 3.1. Clinical Features

A total of 102 patients were admitted with dental infections, presenting with pain in 102 (100%), swelling in 101 (99%), trismus in 41 (40.2%), and dysphagia in 28 (27.4%) patients (Table 1).

### 3.2. Vital Signs

Abnormal values for vital signs included elevated body temperature (>37 °C) in 21 (21%), tachycardia in 25 (24.8%), tachypnoea in 8 (9.3%), hypertension (140 mmHg plus systolic blood pressure) in 28 (29.2%), and higher blood sugar level (12 mmole or more) in 3 (4.8%) patients, who were all diabetic (Table 1).

### 3.3. Time of Presentation

**Figure 1:**
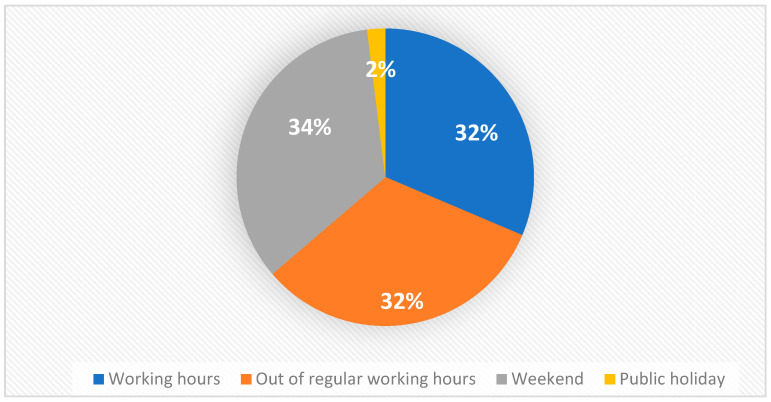
Percentages of dental infections based on the time of presentations.

Most of the patients, 70 in total (68%), requiring hospitalisations presented on weekends, out of regular working hours, and public holidays (Figure 1).

### 3.4. Previous Treatment

**Figure 2:**
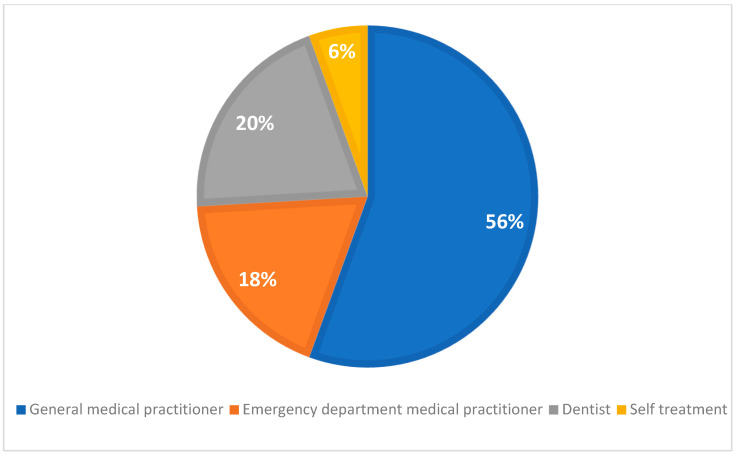
Percentage of patients with only empirical antibiotics prescription prior to presentations (54/102).

Previous treatments related to the presentations were reported in 68 (66.7%) patients; notably, empirical antibiotics prescriptions were given to 54 (52.9%) patients predominantly by medical practitioners (74%) (Table 2) (Figure 2). Most patients (40) (78%) received prescriptions of antibiotics within 7 days, and 11 (21.6%) patients had taken antibiotics more than 7 days prior to presentation.

### 3.5. Demographics

**Figure 3:**
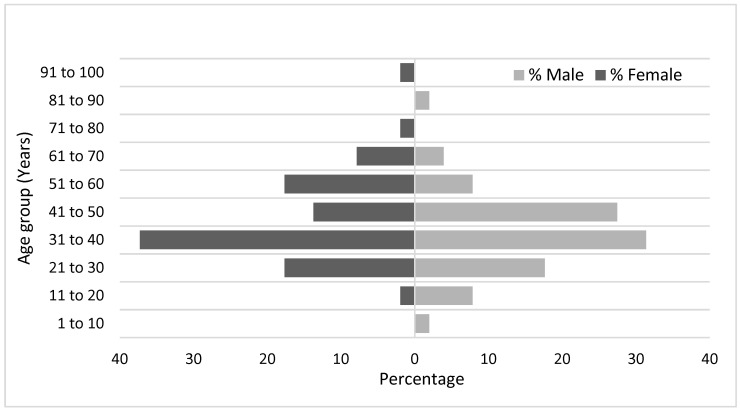
Patient distribution based on gender and age.

There were no gender differences. The mean age of the study group was 40.1 years, while the highest presentations were reported in the thirties in both genders (Figure 3).

### 3.6. Comorbidities

Smoking was reported in 53 (52)% of patients. In total, 52 patients (51%) reported medical comorbidities before admission; 25 (34.2%) patients reported mental health issues, 17 (23.3%) patients reported the use of illicit drug use, 5 (6.8%) patients reported diabetes mellitus, 4 (5.5%) patients reported hepatitis C, and 10 (13.7%) patients reported allergy to penicillin (Table 3).

### 3.7. Teeth and Facial Space Involvement

Sixty-four percent of patients presented with 2 or more facial space involvement: buccal space (44.9%), canine space (17.6%), and submandibular space (13.6%) (Table 4). Dental caries was the main aetiological factor (62.7%), and the common teeth involved were molar teeth (40.9%) (Table 5).

### 3.8. White Blood Cell Counts and C-Reactive Protein

Elevated values of white blood cell count was reported in 51.6% of cases (more than 11 × 10^9^/L) and C-reactive protein in 78% of cases (more than 10 mg/L) (Table 5).

### 3.9. Imaging Modality

The most common imaging modality was orthopantomogram (89.2%), while contrast computed tomography scan (73.5%) was used to investigate inflammatory changes (100%) and collection of abscesses (25.3%) (Table 5).

### 3.10. Management

Surgical management was performed in 78 (76.5%) cases, including 63 (61.8%) extractions mainly under local anaesthesia and 44 (56.4%) in the dental clinic, while incision and drainage were mainly intraoral and required in 38 (37.2%) patients. Augmentin was administered as the main antibiotic for both intravenous in 71 (69.6%) patients in the hospital and discharge prescriptions were given to 77 (79.4%) patients (Table 6).

### 3.11. Hospitalisations

The total length of hospital stay was 271 days (mean: 2.7, SD: 1.6), including 15 (5.9%) days in the intensive care unit (Table 6). The associated complications (8.8%) were mainly related to airway compromise in five patients (4.9%), with no long-term complications and mortality (Table 6).

### 3.12. Microbiology

Specimens were collected for microbiological examinations in 31 (30.4%) patients with positive culture growth in 19 (61.1%) specimens, predominantly normal oral flora, Gram-positive polymorphs, and cocci (Table 7).

## 4. Discussion

Dental infections requiring hospitalisation not only present with significant morbidity and occasional mortality but also carry significant financial implications for both patients and healthcare systems. Our study sheds light on various aspects of these infections, including their temporal patterns, patient demographics, clinical characteristics, diagnostic approaches, management strategies, and clinical outcomes.

Temporal patterns of presentation reveal a concerning trend, with a majority of patients with dental infections (68%) presenting to public hospitals during weekends, out of regular working hours, and on public holidays. Similar patterns have been observed elsewhere, with likely causes attributed to factors such as the limited availability of dentists during these hours, patient education, anxiety, and cost concerns [20]. This distribution can lead to increased strain on emergency healthcare services, potentially leading to longer wait times and preventable hospitalisations. Australia’s national health insurance scheme, Medicare, excludes the adult population from dental care, leaving a gap in access to essential services [21]. Consequently, adult patients with dental infections seek dental consultations from general practitioners or present to public hospitals for treatment. In this study, a large proportion of patients (52.9%) received empirical antibiotic prescriptions, mainly from general medical practitioners (74%), a finding that is consistent with previous studies (33–75%) [22,23]. The prevalent prescription of antibiotics without active dental treatment by general practitioners is alarming [24]. The inappropriate use of empirical antibiotic prescription without active dental interventions can lead to antimicrobial resistance, increased morbidity and mortality, potential adverse reactions, drug interactions, and increased healthcare expenditure [25,26].

This study observed an equal distribution of gender presentations, which contrasts with the findings from previous Australian studies reporting predominantly male populations with dental infections requiring hospitalisations. However, the highest presentations of dental infections occurring in the fourth decade of life, a demographic often actively involved in the workforce, aligns with both the Australian and global data [7,27,28,29,30,31,32]. The prevalence of comorbid conditions such as smoking, illicit drug use, and mental health issues is consistent with findings from other studies [11]. Individuals with these conditions are at elevated risk of dental caries and periodontal disease and are less likely to seek preventative dental care [33].

The clinical presentations of dental infections characterised by severe pain and immediate facial swelling seem to be the main trigger for patients seeking emergency services, although trismus and dysphagia were reported in 40% and 27% of patients, respectively, which was mainly associated with those originating in mandibular teeth. Abnormal values of vital signs were observed in a relatively smaller proportion of patients presenting with dental infections (Table 1), indicating reduced systemic involvement and disease severity. The number of patients presenting with abnormal white blood cell counts (52.7%) and C-reactive protein (78.1%) reported in this study were comparable with other reports [34].

The majority of dental infections were of pulpal origin followed by periodontitis and pericoronitis, which is consistent with findings from other studies [11]. Notably, while all teeth were involved; molar teeth (40.9%), including third molar teeth (9.8%), were predominantly presented. The anatomical position of lower molar teeth leading to trismus, dysphagia, and airway complications observed aligns with previous studies [1,35]. The spread of the dental infection frequently involves multiple spaces, notably buccal space, canine space, and submandibular space, resulting in facial swelling and triggering emergency hospital presentation [1]. In this study, contrast computed tomography was employed in 75 (73.5%) patients, revealing a frank collection of abscesses in 19 (25.3%) patients, who mostly required procedures under general anaesthesia in a hospital setup. A significant proportion of inpatients (56.4%) were referred to the nearby teaching dental hospital, ‘Nepean Centre for Oral Health’, for procedures under local anaesthesia, which provides dental services during regular working hours on weekdays to eligible patients. Although the antibiotic of choice was Augmentin, a change to definitive antibiotics was required in five patients due to resistant bacterial strains.

The mean length of hospital stay (M = 2.7, SD = 1.6, days) and total intensive care unit days (15 days) were both less than those reported in our previous study [11]. Likewise, the absence of mortality and relatively fewer complications, primarily related to airway compromise resulting from dental infections, represents a favourable outcome compared to previous reports [11]. Despite favourable clinical outcomes observed in our study, the financial impact of odontogenic infections remains a significant concern.

The limitations of the study include a small sample size, a single-centre study, and the sample representing only the population of the Nepean blue mountains local health district. There is some heterogenicity in data reporting as some data are missing or not reported for some patients in patients’ electronic records. Due to these limitations, the current study may not represent the true picture of dental infections requiring hospitalisations in Australia. Nonetheless, to the best of our knowledge, this is the first study in New South Wales, the most populated state of Australia, investigating the presentations of dental infections in the human population of all age groups requiring hospitalisations.

## 5. Conclusions

In conclusion, this study provides important information about the characteristics, management, and trends of dental infections leading to potentially preventable hospitalisations, which continue to be a significant burden on the public healthcare system. A notable finding was the highest presentations of dental infections requiring hospitalisations to the emergency department of Nepean hospital on weekends, outside regular working hours, and public holidays, predominantly requiring dental interventions under local anaesthesia. The provision of emergency or on-call dental services on weekends, outside regular working hours, and on public holidays may help reduce the number of potentially preventable hospitalisations and the length of hospital stay. Although this study provides some insight into dental infections requiring hospitalisations in the limited Australian literature, more nationwide and multicentre studies with a larger population reporting on all variables and statistical co-relations between various characteristics and length of hospital stay are required to help guide preventative policies.

## Figures and Tables

**Table 1:** Clinical features and vital signs associated with dental infections.

Pain (*n* = 102)		101 (99%)
Facial swelling (*n* = 102)		102 (100%)
Trismus (*n* = 102)		41 (40.2%)
Dysphagia (*n* = 102)		28 (27.4%)
Respiratory rate (*n* = 86) (number of breaths per minute)	M = 18.5, SD = 2.3, Mdn = 18, R = 14–31	
	Breaths per minute > 20	8 (9.3%)
Oxygen saturation (%) (*n* = 100)	M = 97.6, SD = 1.6, Mdn = 98, R = 95–100	
Pulse rate (*n* = 101)	M = 91.2, SD = 16.4, Mdn = 89, R = 61–165	
	Pulse rate > 100	25 (24.8%)
Systolic blood pressure (mmHg) (*n* = 96)	M = 132.5, SD = 17.1, Mdn = 130, R = 102–189	
	Systolic blood pressure > 140	28 (29.2%)
Diastolic blood pressure (*n* = 95) (mmHg)	M = 82, SD = 10.1, Mdn = 82, R = 52–114	
	Diastolic blood pressure >90	13 (13.7%)
Mean arterial pressure (*n* = 94) (mmHg)	M = 97.6, SD = 12.5, Mdn = 98, R = 39.4–125	
	Mean arterial pressure > 100	39 (41.5%)
Temperature (°C) (*n* = 100)	M = 36.6, SD = 0.7, Mdn = 36.6, R = 35.5–39.2	
	Temperature 37.1–38	18 (18%)
	Temperature > 38	3 (3%)
Random blood sugar level (mmol/L) nondiabetic patients (*n* = 62)	M = 5.3, SD = 0.7, Mdn = 5.2, R = 3.7–7.2	
Random blood sugar level (mmol/L) diabetic patients (*n* = 4)	M = 15.7, SD = 8.7, Mdn = 17.2, R = 5–23.5	

M, mean; SD, standard deviation; Mdn, median; R, range.

**Table 2:** Treatments prior to presentations.

Previous treatment		68 (66.7%)
Antibiotics *		54 (52.9%)
Time of prescription (*n* = 51)	More than a week	11 (21.6%)
	Less than a week	40 (78.4%)
Two or more than two antibiotic prescriptions		11 (20.4%)
Dental treatment	Extractions * +/− Antibiotics	12 (17.6%)
	Pulp extirpations +/− Antibiotics	8 (11.8%)
Types of antibiotics prescribed prior to presentations **	Augmentin	19
	Amoxicillin	15
	Amoxicillin plus Metronidazole	5
	Cefalexin	3
	Augmentin plus metronidazole	1
	Cephalexin plus metronidazole	1
	Clindamycin	1
	Clindamycin plus metronidazole	1
	Flucloxacillin plus metronidazole	1
	metronidazole	1

* Some patients in this group received treatments from medical practitioners (antibiotics) as well as dental practitioners (extractions). ** Some antibiotics’ names were not reported in patients’ clinical records.

**Table 3:** Demographic information and associated comorbidities.

Gender	Male	51 (50%)
	Female	51 (50%)
Age (Years)	M = 40.1, SD = 11.5, Mdn = 37.5, R = 6–93	
Country of Birth	Australia	84 (82.4%)
	Overseas	18 (17.6%)
Aboriginality status	Aboriginal or Torres straits islanders	7 (6.9%)
Smoking status	Smokers	53 (52%)
	Non-smokers	49 (48%)
Smoking Frequency (cigarettes per day)	M = 11.6, SD = 7.1, Mdn = 10, R = 3–30	
Co-morbidity (*n* = 52)	Mental health issues	25 (34.2%)
	Drug use	17 (23.3%)
	Allergy to penicillin	10 (13.7%)
	Diabetes mellitus	5 (6.8%)
	Hepatitis C	4 (5.5%)
	Cancer	2 (2.7%)
	Fatty liver	2 (2.7%)
	Myocardial infarction	1 (1.7%)
	Cardiomyopathy	1 (1.7%)
	COPD	1 (1.7%)
	Epilepsy	1 (1.7%)
	Osteoporosis	1 (1.7%)
	Crohn’s disease	1 (1.7%)
	Heavy alcohol	1 (1.7%)
	Hypothyroid	1 (1.7%)
BMI (*n* = 89)	Mean = 29.9, SD = 9, Mdn = 29.1, R = 16.6–76	

M, mean; SD, standard deviation; Mdn, median; R, range; COPD, chronic obstructive pulmonary disease; BMI, body mass index.

**Table 4:** Facial space involvement.

1 space (*n* = 37) (36%)	Buccal				27
	Canine				8
	Sub-masseteric				1
	Lower lip				1
2 spaces (*n* = 40) (39%)	Space 1	Space 2			
	Buccal	Submandibular			11
	Buccal	Canine			8
	Buccal	Maxillary sinus			7
	Canine	Upper lip			3
	Canine	Orbital			3
	Buccal	Masticator			2
	Buccal	Palatal			1
	Buccal	Sublingual			1
	Buccal	sub masseteric			1
	Submandibular	Submental			1
	Submandibular	Masticator			1
	Submental	Lower lip			1
3 spaces (*n* = 21) (21%)	Space 1	Space 2	Space 3		
	Canine	Buccal	Maxillary sinus		4
	Canine	Buccal	Upper lip		4
	Buccal	Submandibular	Masticator		2
	Submandibular	Masticator	Pharyngeal		2
	Buccal	Sub-masseteric	Pharyngeal		1
	Buccal	Sub-masseteric	Submandibular		1
	Buccal	Sub-masseteric	Masticator		1
	Buccal	Sub-masseteric	Maxillary sinus		1
	Buccal	Sublingual	Submandibular		1
	Buccal	Sublingual	Submental		1
	Buccal	Maxillary sinus	Orbital		1
	Buccal	Canine	Orbital		1
	Sublingual	Submandibular	Masticator		1
≥4 spaces (*n* = 4) (4%)	Space 1	Space 2	Space 3	Space 4	
	Buccal	Sublingual	Submandibular	Pharyngeal	1
	Sublingual *	Submandibular *	Submental	Sub-masseteric	1
	Canine	Buccal space	Maxillary sinus	Orbital	1
	Buccal	Submandibular	Masticator	Pharyngeal	1
Total Patients					102

* Bilateral involvement in Ludwig’s angina.

**Table 5:** Aetiology and investigations.

Aetiology (*n* = 102)	Dental caries (*n* = 64)	Un-restored caries	46 (45.5%)
		Retained root caries	14 (13.9%)
		Restored caries	3 (3%)
		Periapical cyst	1 (1%)
	Periodontal origin (*n* = 10)	Pericoronitis	6 (5.9%)
		Periodontal abscess	4 (4%)
	Post extraction		13 (12.9%)
	Post pulp extirpation		8 (7.9%)
	Failed root canal treatment		4 (4%)
	Occlusal wear		2 (2.0%)
	Tooth fracture		1 (1%)
Jaw involvement	Upper jaw (Maxilla)		49 (48%)
	Lower jaw (Mandible)		53 (52%)
Jaw side involvement	Right		50 (49%)
	Left		52 (51%)
Teeth involvement (Some cases had multiple teeth involved)	Anterior teeth	Incisor teeth	19 (15.6%)
		Canine teeth	13 (10.6%)
	Premolar teeth		39 (32%)
	1st and 2nd molar teeth		38 (31.1%)
	3rd molar teeth		12 (9.8%)
	Deciduous teeth		1 (0.8%)
Investigations	WBC (*n* = 91) (value × 10^9^/L)	M = 11.2, SD = 3.4, Mdn = 11.2, R = 1.5–20.8	
		WBC < 4	1 (1.1%)
		WBC 4 to 11	43 (47.2%)
		WBC > 11	47 (51.6%)
	CRP (*n* = 73) (mg/L)	M = 47.7, SD = 40.6, Mdn = 35, R = 3–186	
		CRP 3 to 10	16 (21.9%)
		CRP 10 to 100	49 (67.1%)
		CRP > 100	8 (10.9%)
	Imaging	Orthopantomogram	91 (89.2%)
		Contrast computed tomography	75 (73.5%)
	Contrast computed tomography results (*n* = 75)	Inflammatory changes	75 (100%)
		Frank abscess collection	19 (25.3%)

WBC, white blood cell count; CRP, C-reactive protein; M, mean; SD, standard deviation; Mdn, median; R, range.

**Table 6:** In-patient management, length of hospital stay, and outcome.

Management	Non-surgical management (antibiotics)		24 (23.5%)
	Surgical management		78 (76.5%)
Anaesthesia	Local anaesthesia (Nepean Centre for Oral Health)		44 (56.4%)
	General anaesthesiia (Nepean hospital)		34 (43.6%)
Surgical options	Extractions		63 (61.8%)
	Incision and drainage (*n* = 38) (37.2%)	Intraoral	33 (32.3%)
		Extraoral	1 (0.9%)
		Combined	4 (3.9%)
	Pulp extirpation		4 (3.9)
Referral to dentist for extraction or pulp extirpation			11 (10.8%)
Intravenous antibiotics (*n* = 102)	Augmentin alone		71 (69.6%)
	Augmentin plus metronidazole		15 (14.7%)
	Benzylpenicillin plus metronidazole		4 (3.9%)
	Clindamycin plus metronidazole		4 (3.9%)
	Clindamycin		3 (2.9%)
	Metronidazole alone		1 (0.9%)
	Ceftriaxone		1 (0.9%)
	Bactrim plus metronidazole		1 (0.9%)
	Cephazolin		1 (0.9%)
	Cephazolin plus metronidazole		1 (0.9%)
Antibiotics on discharge (*n* = 97)	Augmentin		77 (79.4%)
	Augmentin plus metronidazole		4 (4.1%)
	Clindamycin		5 (5.1%)
	Clindamycin plus metronidazole		3 (3.1%)
	Amoxicillin plus metronidazole		2 (2%)
	Benzylpenicillin plus metronidazole		2 (2%)
	Trimethoprim/sulfamethoxazole plus metronidazole		1 (1%)
	Metronidazole		1 (1%)
	Cephalexin		1 (1%)
	Cephalexin plus metronidazole		1 (1%)
Length of hospital stay (days)	Total days		271
	Ward days		256 (94.5%)
	Intensive care unit days		15 (5.9%)
	M = 2.7, SD = 1.6, Mdn = 2, R = 1–8		
Complications	Airway-related complications		5 (4.9%)
	Eye-related complications (blurred vision and diplopia)		1 (0.9%)
	Osteomyelitis		1 (0.9%)
	Severe hypotension		1 (0.9%)
	Hardware infection		1 (0.9%)

M, mean; SD, standard deviation; Mdn, median; R, range.

**Table 7:** Microbiological culture results of dental infections.

Microbiological Studies	Swab culture	31 (30.4%)
	Blood culture	1 (0.1%)
	Polymerase chain reaction	2 (2%)
Culture Growth	No Growth	12 (38.7%)
	Growth	19 (61.3%)
Microorganisms Isolated	Normal flora *	12
	*Streptococcus Milleri group*	4
	*Candida*	4
	*Staphylococcus aureus*	2
	*Staphylococcus epidermis*	1
	*Lactobacilli*	1
	*Streptococcus oralis*	1
	*Viridin group of streptococci*	1
	*Yeast cells*	1
Susceptibility test (*n* = 3)	*Staphylococcus aureus*	2
	*Strep Milleri group*	1
	Resistance to penicillin	2
Gram staining (*n* = 14)	*Gram + polymorphs*	14
	*Gram + Cocci*	10
	*Gram + Rods*	5
	*Gram − Rods*	1

* Normal bacterial oral microflora [19].

## Data Availability

The raw data supporting the conclusions of this article will be made available by the authors on request.
